# A Structurally Characterized *Lycium barbarum* Polysaccharide Protects Against Retinal Degeneration Through Nrf2 Activation and NF-κB/NLRP3 Suppression

**DOI:** 10.3390/antiox15091055

**Published:** 2026-08-24

**Authors:** Yijing Yang, Shuting Yin, Ying Deng, Li Xiao, Yasha Zhou, Jing Lu, Qinghua Peng, J. Arjuna Ratnayaka

**Affiliations:** 1 Key Laboratory for Quality Evaluation of Bulk Herbs of Hunan Province, Hunan University of Chinese Medicine, Changsha 410208, China; 004761@hnucm.edu.cn (Y.Y.); styin@hnucm.edu.cn (S.Y.); 004762@hnucm.edu.cn (Y.D.); xl_646742655@hnucm.edu.cn (L.X.); yashazhou@hnucm.edu.cn (Y.Z.); 004026@hnucm.edu.cn (J.L.); 2 School of Clinical and Experimental Sciences (CES), Faculty of Medicine, University of Southampton, MP 806, Tremona Road, Southampton SO16 6YD, UK; 3 Institute of Ophthalmology and Otolaryngology of Chinese Medicine, Changsha 410208, China; 4 Changsha Centre for Innovation in Traditional Chinese Medicine Techniques for the Prevention and Treatment of Retinal Diseases and Visual Function Protection, Changsha 410208, China

**Keywords:** *Lycium barbarum* polysaccharide, structurally defined fraction, retinal protection, oxidative stress, inflammation, Nrf2, NF-κB

## Abstract

Retinal degeneration is characterized by progressive photoreceptor loss driven by oxidative stress and chronic inflammation, yet effective mutation-independent therapeutic strategies remain limited. *Lycium barbarum* polysaccharides (LBPs) possess antioxidant and anti-inflammatory activities; however, the structural features responsible for their retinal protective effects remain poorly defined. In this study, crude LBP was fractionated by DEAE-cellulose ion-exchange chromatography, and the most bioactive fraction, LBPF2, was identified using H_2_O_2_-injured 661W cone photoreceptor-like cells. LBPF2 was subsequently characterized by high-performance anion-exchange chromatography, SEC-MALLS-RI, GC–MS methylation analysis, and one- and two-dimensional NMR spectroscopy, and its protective effects were evaluated in H_2_O_2_-treated 661W cells and rd10 mice. LBPF2 was identified as a homogeneous glucose-rich polysaccharide with an average molecular weight of approximately 41 kDa and a backbone mainly composed of →4)-α-D-Glcp-(1→ and →4,6)-α-D-Glcp-(1→ residues. LBPF2 reduced oxidative stress, inflammation, and apoptosis in H_2_O_2_-treated 661W cells, preserved retinal morphology, improved electroretinographic responses and partial retention of rhodopsin immunoreactivity relative to untreated rd10 mice, and restored redox homeostasis in rd10 mice. Pharmacological inhibition of Nrf2 using ML385 attenuated these protective effects, supporting the involvement of Nrf2/HO-1 signaling. Collectively, these findings identify LBPF2 as a structurally characterized neuroprotective polysaccharide that mitigates retinal degeneration through coordinated regulation of oxidative stress and inflammation and highlight its therapeutic potential for retinal degenerative diseases.

## 1. Introduction

Retinal degeneration, including inherited retinal dystrophies such as retinitis pigmentosa (RP), remains a major cause of irreversible visual impairment worldwide. Although remarkable progress has been achieved in gene-specific therapies, effective mutation-independent therapeutic strategies applicable to most patients remain limited [1,2]. *Lycium barbarum* L. has long been consumed as both a traditional food and a medicinal plant, and its polysaccharide fraction is widely regarded as one of its principal bioactive constituents [3,4]. Over the past decade, *Lycium barbarum* polysaccharides (LBPs) have attracted increasing attention because of their antioxidant, immunomodulatory, neuroprotective, and metabolic regulatory properties [5,6]. Importantly, accumulating evidence indicates that the biological performance of LBPs is highly dependent on extraction method, purification strategy, monosaccharide composition, molecular size, glycosidic linkage pattern, and chain conformation [5,6,7]. As a result, crude LBP preparations may obscure the contribution of the most bioactive subfractions, and fraction-level structural definition has become increasingly important for interpreting the functionality of goji-derived ingredients [8,9].

The retina represents an attractive target for evaluating the therapeutic potential of LBPs because retinal degeneration is driven by persistent oxidative stress and chronic inflammation. Although inherited retinal degeneration is genetically heterogeneous, oxidative stress, inflammation, and progressive photoreceptor loss are widely recognized as convergent pathogenic features across multiple disease forms [1,10,11]. The retina is particularly vulnerable to redox imbalance because of its high oxygen consumption, abundance of polyunsaturated lipids, intense mitochondrial demand, and continuous exposure to photo-oxidative stress [12,13,14]. Once oxidative injury is initiated, inflammatory signaling further amplifies tissue damage and accelerates structural and functional decline [15,16,17]. In this framework, nuclear factor erythroid 2-related factor 2 (Nrf2) serves as a major endogenous antioxidant regulator [18,19], whereas nuclear factor kappa B (NF-κB) coordinates inflammatory gene expression and contributes to inflammasome priming [20,21,22]. These divergent pathways are therefore highly relevant for evaluating food-derived polysaccharides with dual antioxidant and anti-inflammatory potential [20,23,24].

Previous studies have already suggested that goji-derived polysaccharides can benefit retinal tissues. LBP has been shown to protect photoreceptors in light-induced retinal degeneration [25] and to improve retinal morphology and function in rd1 mice during photoreceptor degeneration [26]. These observations support the concept that goji polysaccharides have retinal protective potential. However, most prior work has relied on crude or broadly defined LBP preparations rather than structurally characterized active fractions. Consequently, it remains unclear which fraction-level features are most closely associated with retinal protection, and whether such effects can be interpreted within a more precise food-function framework.

In the present study, crude LBP was fractionated and screened in hydrogen peroxide (H_2_O_2_)-injured 661W photoreceptor-like cells, leading to the identification of LBPF2 as the most bioactive fraction compared to LBPF1. LBPF2 was then subjected to detailed structural characterization by high-performance anion-exchange chromatography, SEC-MALLS-RI, GC-MS methylation analysis, and one- and two-dimensional NMR spectroscopy, followed by functional evaluation in H_2_O_2_-treated 661W cells and rd10 mice. By integrating fraction screening, comprehensive structural characterization, and mechanism-oriented biological validation, this study aimed to identify a structurally defined polysaccharide responsible for retinal protection and to establish a clearer structure–activity relationship for *Lycium barbarum* polysaccharides in retinal degeneration.

## 2. Materials and Methods

### 2.1. Reagents and Chemicals

Crude *Lycium barbarum* polysaccharides (LBPs, C0218) were purchased from Duly Bio-Technology Co., Ltd. (Nanjing, China). Monosaccharide standards—including fucose (Fuc, 2438-80-4), rhamnose (Rha, 10030-85-0), arabinose (Ara, 5328-37-0), galactose (Gal, 5328-37-0), glucose (Glc, 50-99-7), xylose (Xyl, 58-86-6), mannose (Man, 3458-28-4), fructose (Fru, 57-48-7), ribose (Rib, 50-69-1), galacturonic acid (Gal-UA, 14982-50-4), glucuronic acid (Glc-UA, 6556-12-3), mannuronic acid (Man-UA, 6814-36-4), and guluronic acid (Gul-UA, 15769-56-9) were obtained from Sigma-Aldrich Co., Ltd. (Shanghai, China). Cell Counting Kit-8 (C0038, Beyotime, Shanghai, China). Mouse TNF-alpha Quantikine ELISA Kit (MTA00B, R&D Systems), Mouse IL-1 beta Quantikine ELISA Kit (MLB00C, R&D Systems, Shanghai, China), and Mouse IL-6 Quantikine ELISA Kit (M6000B, R&D Systems, Shanghai, China). Nrf2 inhibitor ML385 (CAS No. HY-100523, MedChemExpress, Shanghai, China), Nrf2 activator Dimethyl fumarate (CAS No. HY-17363, MedChemExpress, Shanghai, China), NF-κB activator (HY-134477, MedChemExpress, Shanghai, China), and NF-κB inhibitor (HY-144765, MedChemExpress). Primary antibodies against Nrf2 (AF0639), HO-1 (AF5393), Caspase-3 (AF6311), NF-κB p65 (AF5006), phospho-NF-κB p65 (AF2006), NLRP3 (DF7438), and β-actin (AF7018) were purchased from Affinity Biosciences (Jiangsu, Changzhou, China) and used at a dilution of 1:1000. HRP-conjugated secondary antibody against mouse IgG (7076S, Cell Signaling Technology, Danvers, MA, USA) was used at a dilution of 1:10,000.

### 2.2. Purification of Lycium barbarum Polysaccharide Fractions

#### 2.2.1. DEAE-Cellulose Ion-Exchange Chromatography

The sample was applied to a DEAE-cellulose column (26 mm × 400 mm), which was sequentially eluted with distilled water at 4 mL/min, followed by 0.1, 0.2, and 0.3 M NaCl. Carbohydrate-containing fractions were monitored using the phenol–sulfuric acid method at 490 nm. Similar chromatographic procedures have been widely employed for the purification of *Lycium barbarum* polysaccharides and related glucan fractions [5,6]. We selected two main peak polysaccharide components for subsequent experiments.

#### 2.2.2. Screening of Bioactive Fractions in 661W Cells

We induced oxidative stress damage in 661W cells, a photoreceptor-like cell line, using hydrogen peroxide as our model. The induction method has been described in our previous studies, where we treated 661W cells with 200 μmol/L H_2_O_2_ for 24 h [27]. LBPF1 and LBPF2 were initially evaluated at concentrations of 10, 20, 40, 80, 160 and 320 μg/mL using the CCK-8 assay. Based on this concentration-screening experiment, 80 and 160 μg/mL were selected as the intermediate and high concentrations, respectively, for subsequent experiments in H_2_O_2_-injured 661W cells. Intracellular reactive oxygen species (ROS) production and Annexin V/PI-defined cell loss were subsequently measured after treatment with these two concentrations. The 320 μg/mL concentration was not used in subsequent mechanistic experiments because it markedly reduced cell viability.

### 2.3. Structural Characterization of LBPF2

#### 2.3.1. Monosaccharide Composition Analysis by HPAEC

Sample extracts were analyzed using High-performance anion-exchange chromatography (HPAEC) on a CarboPac PA-20 column (3mm × 150 mm) with a PAD (Dionex ICS 5000+, Thermo Fisher Scientific, Sunnyvale, CA, USA). Flow rate: 0.5 mL/min; injection volume: 5 μL. Solvents included: A (ddH_2_O), B (0.1 M NaOH), C (0.1 M NaOH + 0.2 M NaAc). Data were collected on ICS5000+, processed with Chromeleon 7.2 CDS.

#### 2.3.2. Molecular Weight Determination by SEC-MALLS-RI

The homogeneity and molecular weight (Mw, Mn, Mw/Mn) of fractions in 0.1 M NaNO_3_ were analyzed using SEC-MALLS-RI with a DAWN HELEOS-II photometer and an Optilab T-rEX detector. The system, equipped with tandem Shodex OH-pak columns at 45 °C and a flow rate of 0.6 mL/min, employed a determined dn/dc value of 0.141 mL/g for the sample solution.

#### 2.3.3. Methylation Analysis by GC–MS

The polysaccharide was methylated in DMSO/NaOH/CH_3_I, then hydrolyzed with 2 M TFA at 121 °C for 1.5 h. The products were reduced with NaBD_4_ and acetylated at 100 °C for 2.5 h. The resulting acetates were analyzed by Gas chromatography-mass spectrometry (GC–MS) (Agilent 6890A-5977B, Santa Clara, CA, USA) using a BPX70 column (30 m × 0.25 mm × 0.25 µm) with helium as the carrier gas (split ratio 10:1). The oven was held at 140 °C for 2 min, then ramped to 230 °C at 3 °C per minute and maintained at that temperature for 3 min. Detection was carried out in SCAN mode (*m*/*z* 50–350).

#### 2.3.4. Scanning Electron Microscopy

The molecular structures of polysaccharides were examined using a scanning electron microscope (Zeiss Merlin Compact, Carl Zeiss AG, Oberkochen, Germany). The samples, coated with a thin gold layer, were placed on the substrate, and images were captured at 1.0 kV with 10,000-fold magnification under high vacuum.

#### 2.3.5. NMR Spectroscopy

The sample was dissolved in 0.5 mL D2O to achieve a final concentration of 40 mg/mL. 1D-Nuclear Magnetic Resonance (NMR) (1H-NMR, 13C-NMR, DEPT135) and 2D-NMR (COSY, NOESY, HMBC, HSQC, and TOCSY) were recorded at 25 °C using a BrukerAVANCE NEO 500 MHz spectrometer system (Bruker, Rheinstetten, Germany).

### 2.4. Cell Culture and Experimental Design

#### 2.4.1. Cell Culture and Establishment of Oxidative Stress Model

The mouse cone photoreceptor-derived 661W cell line [28] was cultured in DMEM supplemented with 10% fetal bovine serum and 1% penicillin–streptomycin at 37 °C in a humidified atmosphere containing 5% CO_2_. 661W cells were exposed to 200 μM H_2_O_2_ for 24 h to induce oxidative stress.

#### 2.4.2. Pharmacological Modulation of Nrf2 and NF-κB Signaling

To investigate the involvement of Nrf2 and NF-κB signaling pathways, pharmacological activators and inhibitors were employed. NF-κB signaling was modulated using NF-κB Activator 2 (5 mM) and the selective inhibitor NF-κB-IN-4 (10 μM). Nrf2 signaling was activated using dimethyl fumarate (DMF, 45 μM), a well-established Nrf2 agonist, and inhibited using ML385 (10 μM), a selective Nrf2 inhibitor. Concentrations were selected based on previous reports demonstrating effective pathway modulation and minimal cytotoxicity [29,30].

### 2.5. Flow Cytometric Analysis of ROS and ELISA

Using hydrogen peroxide-induced 661W cells as the study subjects, measurements were taken after 24 h of intervention with LBP at different concentrations. Intracellular ROS production and apoptosis/cell loss were quantified by flow cytometry.

Inflammatory cytokines, including tumor necrosis factor-α (TNF-α), interleukin-1β (IL-1β), and interleukin-6 (IL-6), were quantified using commercially available ELISA kits (Elabscience, Wuhan, China) according to the manufacturer’s instructions. Cell culture supernatants were collected after treatment and centrifuged at 2000 rpm for 5 min to remove debris before analysis.

To evaluate oxidative stress, malondialdehyde (MDA), superoxide dismutase (SOD), and glutathione (GSH) levels were determined using corresponding ELISA kits following the manufacturer’s protocols. Absorbance was measured using a microplate reader, and concentrations were calculated based on standard calibration curves.

### 2.6. Western Blotting (WB)

Total proteins were extracted using RIPA buffer. Equal amounts of protein were separated by SDS-PAGE, transferred to PVDF membranes, incubated with primary antibodies (1:1000) overnight at 4 °C, followed by HRP-conjugated secondary antibodies (1:10,000). Protein bands were visualized using enhanced chemiluminescence and quantified using ImageJ software (ImageJ2x 2.1.4.7).

### 2.7. Animal Experiments

Healthy SPF four-week-old C57BL/6J mice with weight ranged from 18g to 20g served as the control group (*n* = 10, 5 males and 5 females). Age- and weight-matched rd10 mice were purchased from The Jackson Laboratory (USA; certificate No. 1911A11353) [31]. Animals were allocated to treatment groups using a random-number table. Procedures and outcome assessments were conducted in a balanced order across groups, and cage placement was arranged to minimise cage-position and measurement-order effects. The group included: model, low-dose LBPF2, high-dose LBPF2, and a combination of Nrf2 activator and NF-κB inhibitor, with each group consisting of 10 mice (5 males and 5 females). Mice were allowed to acclimatise to the SPF animal facility for 7 days before randomisation and treatment.

Mice were housed in the SPF barrier facility of the Experimental Animal Center of Hunan University of Chinese Medicine under controlled temperature, humidity, ventilation, and a 12 h/12 h light–dark cycle, with free access to food and water. Mice in the control group received 0.2 mL of saline daily. rd10 mice were randomly assigned to the model group, low-dose LBPF2 group, high-dose LBPF2 group, and a group treated with Nrf2 activator (Dimethyl fumarate, 45 μM) plus NF-κB inhibitor (NF-κB-IN-4, 10 μM). All treatments were administered once daily for four weeks by oral intragastric gavage at a fixed volume of 0.2 mL per mouse. Wild-type and untreated rd10 mice received 0.2 mL of vehicle through the same route. The low-dose LBPF2 group received 0.2 mL of an 80 μg/mL LBPF2 solution, corresponding to 16 μg per mouse per day. Based on the recorded body-weight range of 18–20 g at the beginning of treatment, this represented a nominal starting dose of approximately 0.80–0.89 mg/kg/day. The high-dose LBPF2 group received 0.2 mL of a 160 μg/mL LBPF2 solution, corresponding to 32 μg per mouse per day or a nominal starting dose of approximately 1.60–1.78 mg/kg/day; The pharmacological comparator solution contained DMF at a final concentration of 45 μM and NF-κB-IN-4 at a final concentration of 10 μM. The two compounds were co-administered once daily in a total volume of 0.2 mL per mouse by oral intragastric gavage. This corresponded to 1.297 μg DMF and 0.645 μg NF-κB-IN-4 per mouse per day. Based on the recorded initial body-weight range of 18–20 g, the nominal starting doses were approximately 0.065–0.072 mg/kg/day for DMF and 0.032–0.036 mg/kg/day for NF-κB-IN-4. The comparator solution was prepared in saline containing 5% DMSO. After 4 weeks of administration, mice were anesthetized with 0.1 mg/mL sodium pentobarbital for retinal electrophysiology (ERG) detection by using the Granzfeld ERG system (Phoenix Research Labs Inc., ERG controller, Serial number 2606, New York, NY, USA). All mice were euthanized following anesthesia, and their eyes enucleated and chorioretinal tissues analyzed by Hematoxylin and eosin (HE) staining, WB, and ELISA. Investigators responsible for animal allocation and gavage were aware of group allocation. Investigators performing ERG recording, histological assessment/ONL measurement, Western blot/ELISA quantification and statistical analysis were blinded to group allocation until analysis was complete. No animals, experimental units or data points were excluded from the analysis.

Retinal sections were subjected to antigen retrieval, blocked with5% bovine serum albumin (BSA) in PBS containing 0.3% Triton X-100 for 1 h at room temperature, and incubated overnight at 4 °C with an anti-rhodopsin primary antibody (Abcam ab5417, dilution 1:500). Sections were subsequently incubated with an Alexa Fluor 488-conjugated secondary antibody (Thermo Fisher A-11034, Shanghai, China; dilution 5 µg/mL) and imaged using a fluorescence microscope under identical exposure, gain and illumination settings. Three standardized retinal regions located at equivalent distances from the optic nerve were analyzed per retina. Background-corrected integrated fluorescence density was quantified using ImageJ.

### 2.8. Bioinformatics Analyses

The bioinformatics database STRING (https://string-db.org/, version 12.0) was utilized (accession date 20 June 2026) to study putative protein–protein interactions in cellular signaling pathways of interest.

### 2.9. Statistical Analysis

Statistical analyses were conducted using GraphPad Prism 9.0 (GraphPad Software, La Jolla, CA, USA). All data are presented as the mean ± standard deviation (SD). Multiple group comparisons were performed using a one-way analysis of Variance (one-way ANOVA) or Kruskal–Wallis’ rank-sum test. For normally distributed data with homogeneous variances, one-way ANOVA followed by Tukey’s multiple-comparisons test was used. The post hoc analysis included direct comparisons between the intermediate-dose (80 μg/mL) and high-dose (160 μg/mL) LBPF2 groups. When parametric assumptions were not satisfied, the Kruskal–Wallis test followed by Dunn’s multiple-comparisons test was applied. Adjusted *p* values are reported for all post hoc comparisons. No linear dose–response model was prespecified. *p* < 0.05 was considered statistically significant. For all in vivo experiments, the experimental unit (n) represents the number of individual animals unless otherwise specified. For retinal histology, ERG, Western blotting, and ELISA analyses, retinal tissues from individual mice were analyzed independently, and each mouse was considered one biological replicate. For in vitro experiments, “n” represents the number of independent biological experiments performed on separate occasions, with each experiment conducted using independently cultured cells.

## 3. Results

### 3.1. Purification of LBP Fractions and Screening of Antioxidant Activity

**Figure 1:**
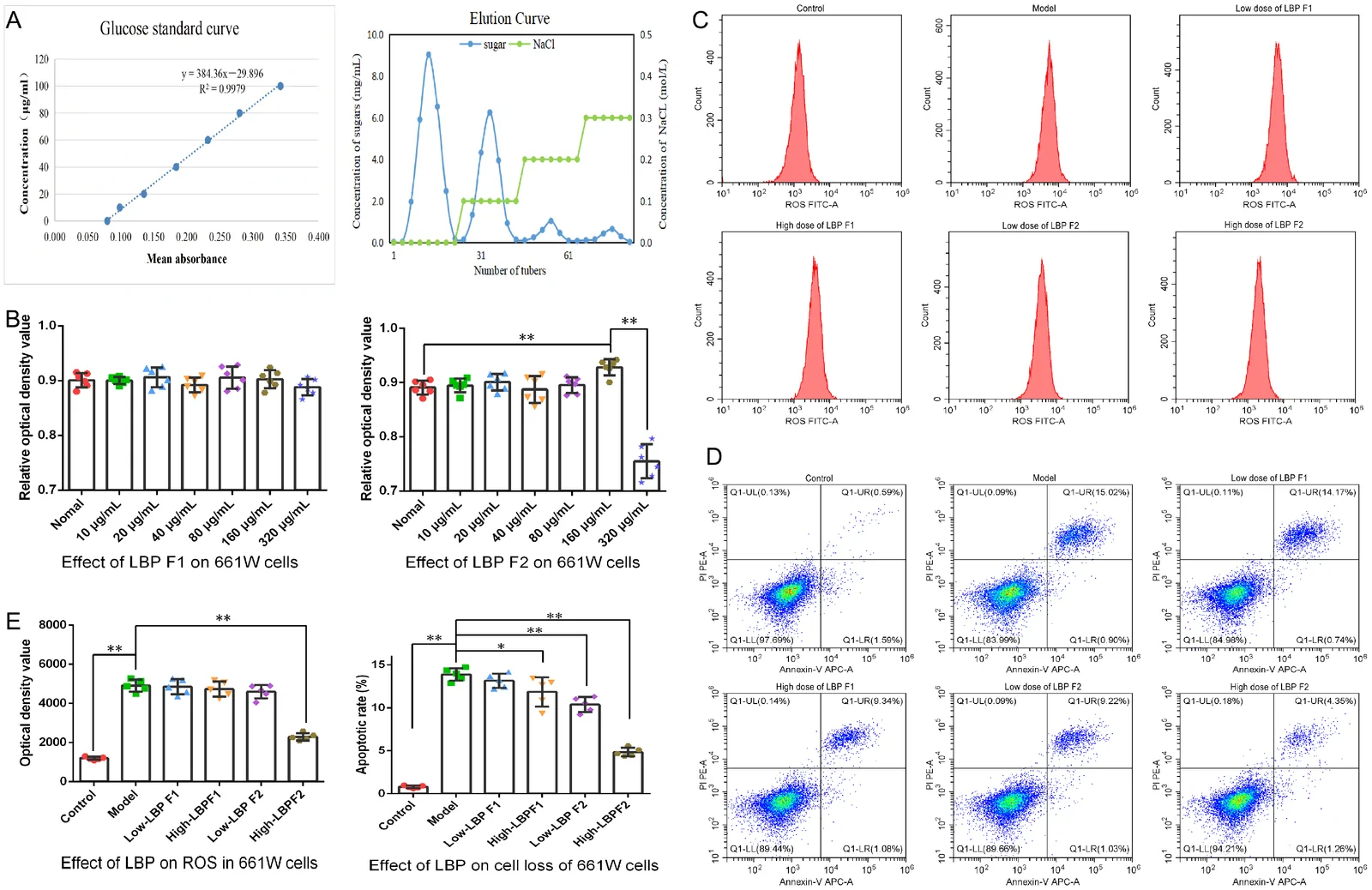
Purification, concentration screening and biological activity of LBP fractions in 661W cells. (**A**) Glucose calibration curve and DEAE-cellulose elution profile. (**B**) Effects of LBPF1 and LBPF2 concentrations ranging from 10 to 320 μg/mL on 661W cell viability. (**C**,**E**) Representative flow-cytometric analysis and quantification of intracellular ROS in H_2_O_2_-injured 661W cells treated with low-dose (80 μg/mL) or high-dose (160 μg/mL) LBPF1 or LBPF2. (**D**,**E**) Representative Annexin V/PI flow-cytometric analysis and quantification of cell loss, green: live cells (PI−); blue: early apoptotic cells; red (sporadic): necrotic cells or doublets. Data are presented as mean ± SD. Statistical comparisons were performed using one-way ANOVA followed by Tukey’s multiple-comparisons test. n = 6, * *p* < 0.05, ** *p* < 0.01.

The total polysaccharide content of the crude *Lycium barbarum* extract was determined using the phenol–sulfuric acid method [32]. A standard calibration curve generated using glucose solutions of known concentrations was used for quantification. Following DEAE-cellulose ion-exchange chromatography, two major polysaccharide fractions were obtained and designated as LBPF1 and LBPF2. The corresponding elution profile revealed two distinct carbohydrate peaks, indicating successful fractionation of the crude polysaccharides (Figure 1A). The purity of LBPF1 and LBPF2 was subsequently determined to be 84.0% and 81.1%, respectively (Table 1).

Initial concentration screening showed that LBPF2 concentrations ranging from 10 to 160 μg/mL did not reduce 661W cell viability, whereas exposure to 320 μg/mL caused a marked decrease in viability (Figure 1B). Among the non-cytotoxic concentrations tested, 160 μg/mL produced the greatest beneficial effect. Direct post hoc comparison between 80 and 160 μg/mL showed that the difference in cell viability was not significant after adjustment for multiple comparisons (adjusted *p* = 0.0062). Based on these findings, 80 and 160 μg/mL were selected as the low and high concentrations for subsequent experiments.

In H_2_O_2_-injured 661W cells, 160 μg/mL LBPF2 markedly reduced intracellular ROS accumulation, whereas 80 μg/mL produced a more moderate effect (Figure 1C,E). Direct comparison between the two LBPF2 concentrations showed that the difference was significant (adjusted *p* < 0.0001). Both concentrations reduced Annexin V/PI-defined cell loss relative to the H_2_O_2_ model group, with a numerically greater reduction at 160 μg/mL. The direct difference between 80 and 160 μg/mL was significant (adjusted *p* = 0.0014) (Figure 1D,E). In comparison, LBPF1 produced substantially weaker effects on ROS accumulation and cell loss.

These findings identify LBPF2 as the more active fraction and 160 μg/mL as the most effective concentration among the non-cytotoxic concentrations tested. However, the decrease in viability observed at 320 μg/mL and the absence of a consistent graded effect across all endpoints indicate a non-linear concentration–response pattern rather than a simple linear dose–response relationship.

### 3.2. Monosaccharide Composition, Molecular Weight Distribution, and Structural Characterization of LBPF2

**Figure 2:**
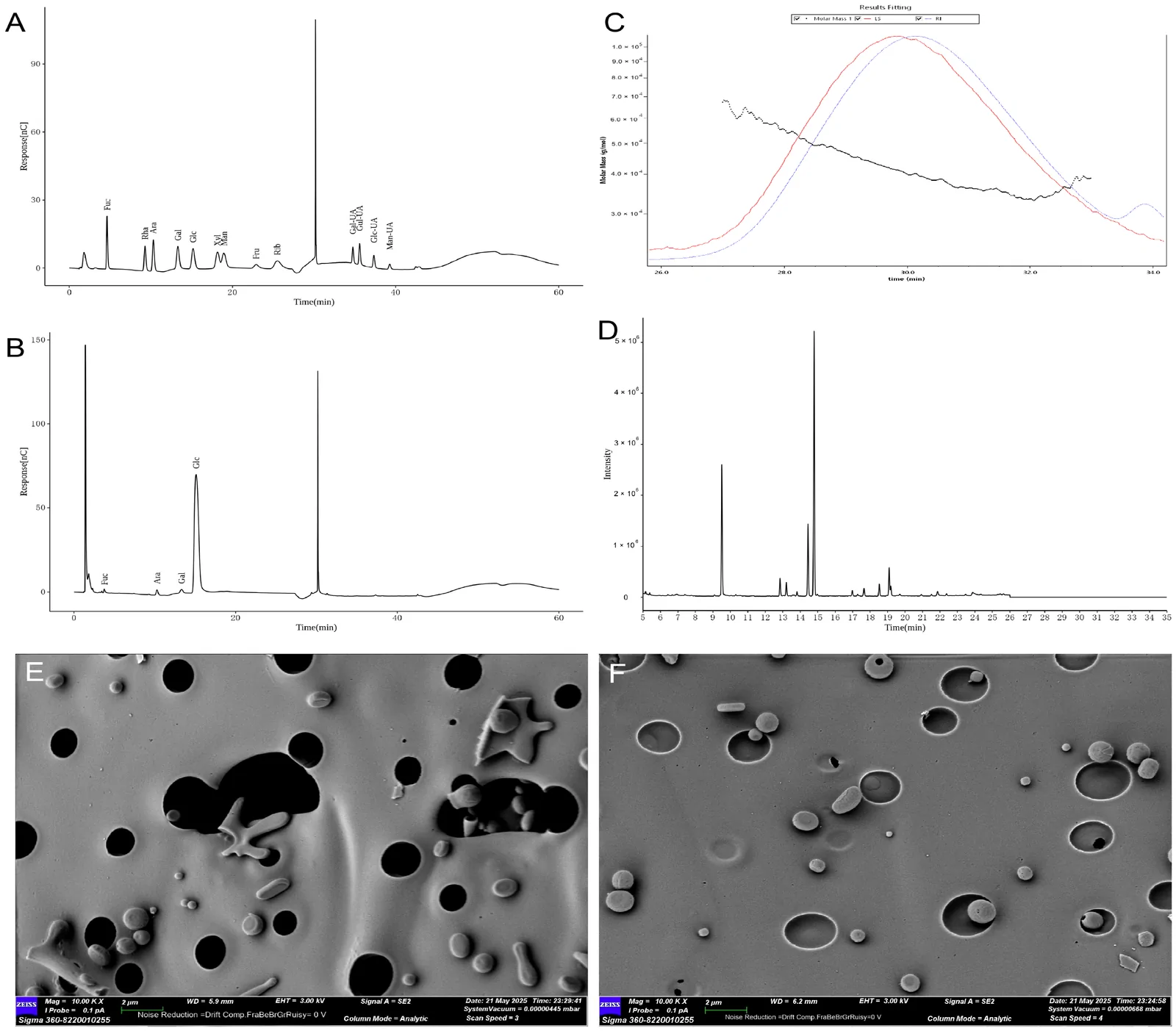
Monosaccharides and polysaccharide analysis of LBPF2. (**A**) Standard solutions of a mixture of 13 monosaccharides. (**B**) Composed of four types of monosaccharides and three unidentified monosaccharides in LBPF2. (**C**) Absolute polysaccharide molecular weight analysis chart: the detected retention time (Time, min) is plotted on the *x*-axis, while the molar mass (g/mol) is plotted on the *y*-axis. (**D**) Total Ion Chromatogram of LBPF2-based analytical sample. (**E**,**F**) scanning electron microscopy of LBPF2 at 10 K magnification.

Given its superior biological activity, LBPF2 was subjected to detailed structural characterization. Monosaccharide composition was first analyzed by high-performance anion-exchange chromatography (HPAEC). Calibration curves were generated using a mixed standard solution containing 13 monosaccharides (Figure 2A).

HPAEC analysis revealed that LBPF2 was predominantly composed of glucose, together with smaller amounts of fucose, arabinose, and galactose. Specifically, the contents of Fuc, Ara, Gal, and Glc were determined to be 2.82, 8.95, 8.34, and 445.16 μg/mg, respectively. In addition, three unidentified peaks were detected in the chromatogram (Figure 2B and Table 2), indicating the presence of minor monosaccharide components.

The molecular weight distribution of LBPF2 was subsequently determined using high-performance gel permeation chromatography coupled with multi-angle laser light scattering. Analysis of the major polysaccharide peak showed a number-average molecular weight (Mn) of 40.309 kDa, a weight-average molecular weight (Mw) of 41.210 kDa, a z-average molecular weight (Mz) of 42.249 kDa, and a peak molecular weight (Mp) of 40.075 kDa. The low polydispersity index (Mw/Mn = 1.022) indicated that LBPF2 is a highly homogeneous polysaccharide fraction (Figure 2C).

To investigate glycosidic linkage patterns, methylation analysis was performed using GC–MS. The major partially methylated alditol acetates identified are summarized in Table 3. The predominant linkage type was 4-linked glucopyranose [4-Glc(p)], accounting for 43.05% of total residues, followed by terminal glucopyranose [t-Glc(p)] (22.94%), 6-linked glucopyranose [6-Glc(p)] (11.82%), and 4,6-linked glucopyranose [4,6-Glc(p)] (5.73%). Minor amounts of 2-, 3-, and branched glucopyranose residues were also detected. These results suggest that LBPF2 possesses a predominantly glucan-based backbone containing both linear and branched structural domains.

The surface morphology of LBPF2 was further examined by scanning electron microscopy. As shown in Figure 2E,F, LBPF2 exhibited an irregular porous structure characterized by circular cavities and heterogeneous particle aggregates, together with adherent rod-like and spherical surface features. The structural characteristics observed by SEM further support the complex macromolecular architecture of LBPF2.

### 3.3. Structural Elucidation of LBPF2 by NMR Spectroscopy

**Figure 3:**
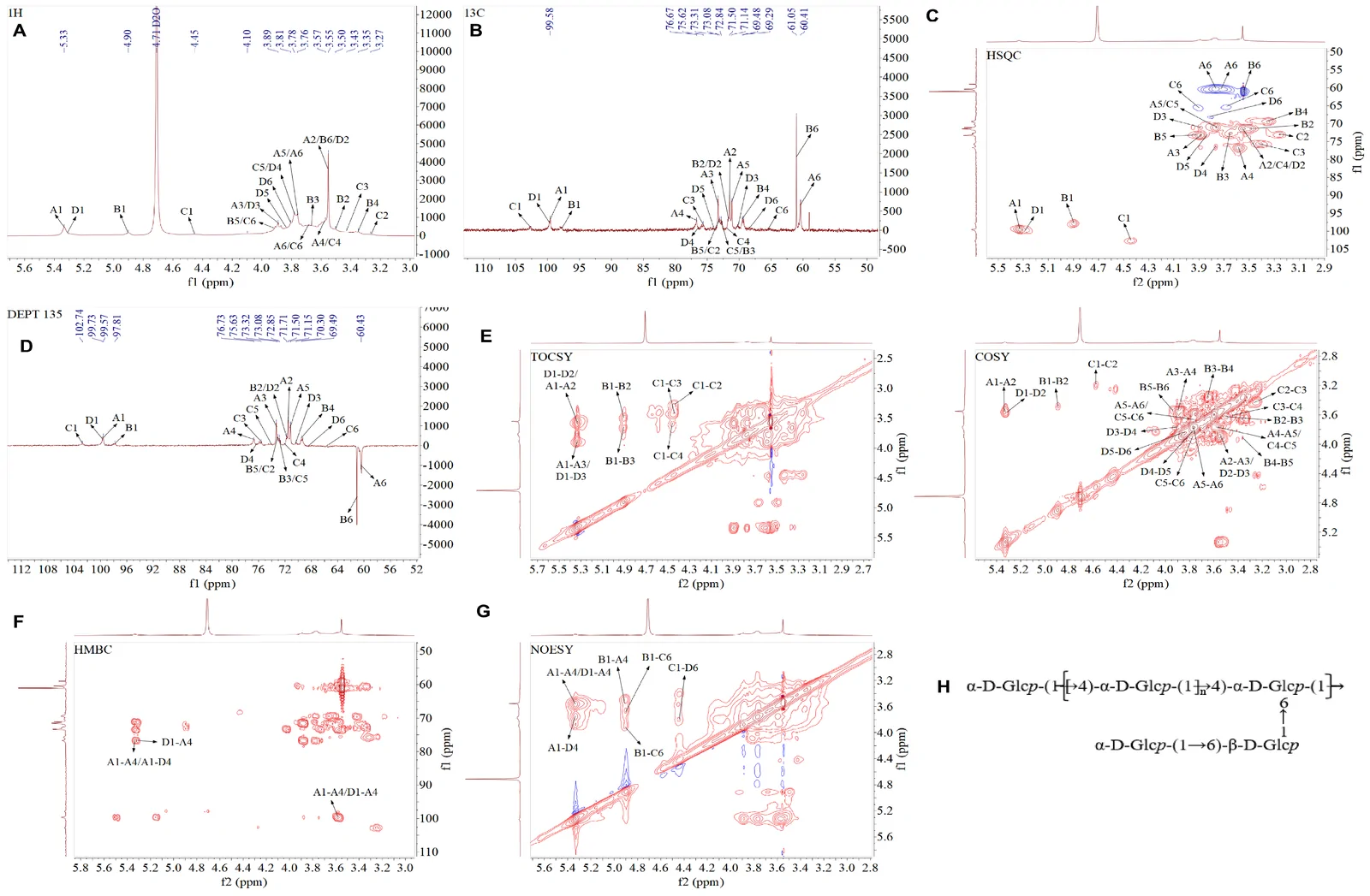
Structural elucidation of LBPF2 by one- and two-dimensional NMR spectroscopy. (**A**) ^1^H NMR spectrum. (**B**) ^13^C NMR spectrum. (**C**) HSQC spectrum showing proton–carbon correlations used for assignment of major glycosyl residues. (**D**) DEPT-135 spectrum used to distinguish methylene, methine, and quaternary carbon signals. (**E**) COSY and TOCSY spectra used for complete proton signal assignments. (**F**) HMBC spectrum showing long-range proton–carbon correlations used to determine glycosidic linkage patterns. (**G**) NOESY spectrum illustrates spatial interactions between adjacent sugar residues. (**H**) Proposed repeating structural unit of LBPF2 based on methylation analysis and NMR spectroscopy.

To further elucidate the fine structure of LBPF2, one-dimensional and two-dimensional NMR analyses were performed. The ^1^H NMR spectrum exhibited characteristic proton signals predominantly distributed between δ 3.0 and 5.5 ppm, indicating the presence of multiple glycosyl residues within the polysaccharide (Figure 3A). Several anomeric proton signals were observed at δ 4.45, 4.90, 5.31, and 5.33 ppm, whereas the corresponding ^13^C NMR spectrum displayed characteristic anomeric carbon signals within the region typical of polysaccharides (Figure 3B). The intense resonance at δ 4.71 ppm was assigned to the residual HDO solvent signal.

Combined analysis of HSQC, COSY, TOCSY, and DEPT135 spectra enabled assignment of four major glycosyl residues, designated as residues A–D (Figure 3C–E). Characteristic anomeric cross-peaks were observed at δ 5.33/99.57, 4.90/97.81, 4.45/102.74, and 5.31/99.73 ppm, respectively. Based on chemical shift characteristics, methylation analysis, and comparison with previously reported polysaccharide structures, residue A was assigned as →4)-α-D-Glcp-(1→, residue B as terminal α-D-Glcp-(1→, residue C as →6)-β-D-Glcp-(1→, and residue D as →4,6)-α-D-Glcp-(1→. Detailed proton and carbon assignments are summarized in Table 4.

To determine the linkage relationships among these residues, HMBC and NOESY spectra were further analyzed (Figure 3F,G). In the HMBC spectrum, clear long-range correlations were observed between H1 of residue A and C4 of residues A and D, as well as between H1 of residue D and C4 of residue A, supporting the existence of a backbone composed of alternating →4)-α-D-Glcp-(1→ and →4,6)-α-D-Glcp-(1→ residues. Consistent with these observations, NOESY analysis revealed significant inter-residue correlations between residues A and D, together with correlations involving terminal α-D-Glcp and →6)-β-D-Glcp residues, indicating that branching occurred at the O-6 position of the →4,6)-α-D-Glcp-(1→ residue.

By integrating methylation analysis with one- and two-dimensional NMR data, the major structural framework of LBPF2 was established (Figure 3H). LBPF2 consists primarily of a backbone formed by →4)-α-D-Glcp-(1→ and →4,6)-α-D-Glcp-(1→ residues, with side chains containing terminal α-D-Glcp-(1→ and →6)-β-D-Glcp-(1→ units attached to the O-6 position of the branching residue. These findings demonstrate that LBPF2 is a highly branched glucan-type polysaccharide with a well-defined structural architecture, providing a structural basis for its biological activity.

### 3.4. LBPF2 Activates Nrf2/HO-1 Signaling and Attenuates Oxidative Stress in 661W Cells

**Figure 4:**
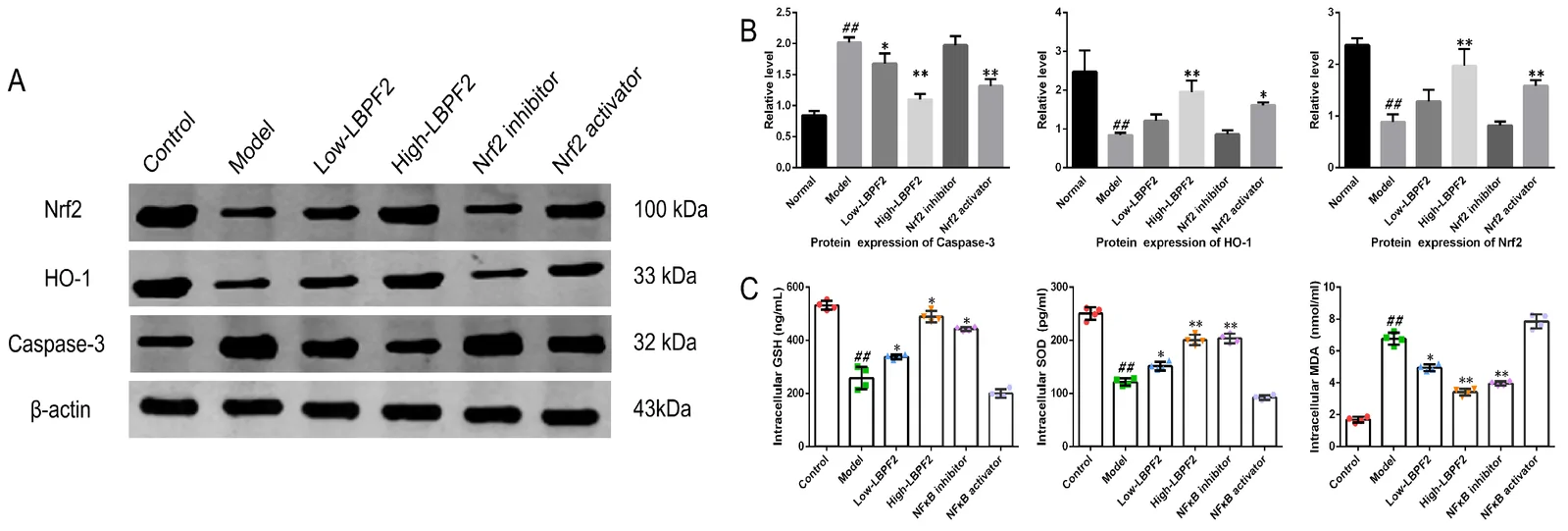
Mechanism of LBPF2 modulation of oxidative stress in 661W cells. (**A**,**B**) WB showed the effects of LBPF2 on protein expression of Nrf2, HO-1 and Casapase-3 (n = 3); (**C**) ELISA assay showed the effects of LBPF2 on oxidative stress markers in H_2_O_2_-induced 661W cells (n = 4). Compared to the control group, ## *p* < 0.01; compared to the model group, * *p* < 0.05, ** *p* < 0.01.

To investigate the antioxidant mechanism underlying the protective effects of LBPF2, the Nrf2/HO-1 signaling pathway was examined in H_2_O_2_-induced 661W cells. Western blot analysis revealed that oxidative stress significantly reduced the expression of Nrf2 and its downstream antioxidant enzyme HO-1. In contrast, LBPF2 treatment markedly increased the protein levels of both Nrf2 and HO-1 while simultaneously decreasing the expression of the apoptotic marker Caspase-3 (Figure 4A).

To further evaluate the intracellular oxidative status, ELISA assays were performed to quantify oxidative stress-related biomarkers. Exposure to H_2_O_2_ significantly increased malondialdehyde (MDA) levels while reducing the antioxidant defenses represented by superoxide dismutase (SOD) and glutathione (GSH). Treatment with LBPF2 effectively reversed these changes by lowering MDA accumulation and restoring SOD and GSH levels (Figure 4B).

These findings indicate that LBPF2 effectively alleviates oxidative stress in photoreceptor-like cells and that activation of the Nrf2/HO-1 pathway is likely involved in mediating its antioxidant effects.

### 3.5. LBPF2 Suppresses NF-κB/NLRP3-Mediated Inflammation in 661W Cells

**Figure 5:**
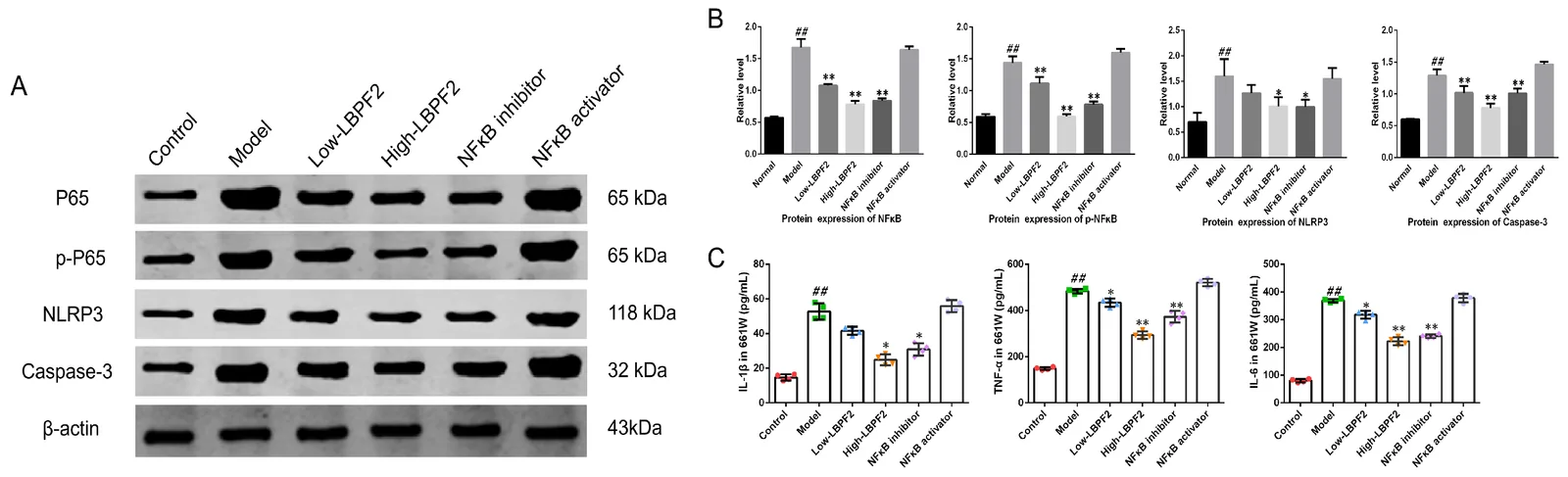
Mechanism of LBPF2 modulation of inflammation in 661W cells. (**A**,**B**) WB showed the effects of LBPF2 on protein expression of NF-κB, p-NF-κB, NLRP3, and Casapase-3 (n = 3); (**C**) ELISA assay showed the effects of LBPF2 on inflammatory markers in H_2_O_2_-induced 661W cells (n = 4). Compared to the control group, ## *p* < 0.01; compared to the model group, * *p* < 0.05, ** *p* < 0.01.

Given the close association between oxidative stress and inflammatory responses, we next investigated whether LBPF2 modulates NF-κB/NLRP3 signaling in H_2_O_2_-treated 661W cells. Western blot analysis demonstrated that oxidative injury markedly increased the expression of NF-κB, phosphorylated NF-κB (p-NF-κB), NLRP3, and Caspase-3. Treatment with LBPF2 significantly reduced the expression of these proteins. Similar inhibitory effects were observed following treatment with a selective NF-κB inhibitor, suggesting that suppression of NF-κB signaling contributes to the anti-inflammatory activity of LBPF2 (Figure 5A).

To further assess inflammatory responses, the levels of TNF-α, IL-1β, and IL-6 were measured by ELISA. H_2_O_2_ exposure significantly elevated the production of these pro-inflammatory cytokines, whereas LBPF2 treatment markedly reduced their levels (Figure 5B).

Together, these results demonstrate that LBPF2 suppresses oxidative stress-induced inflammatory responses in photoreceptor-like cells through inhibition of the NF-κB/NLRP3 signaling pathway.

### 3.6. Inhibition of Nrf2 Attenuates the Protective Effects of LBPF2 in H_2_O_2_-Induced 661W Cells

**Figure 6:**
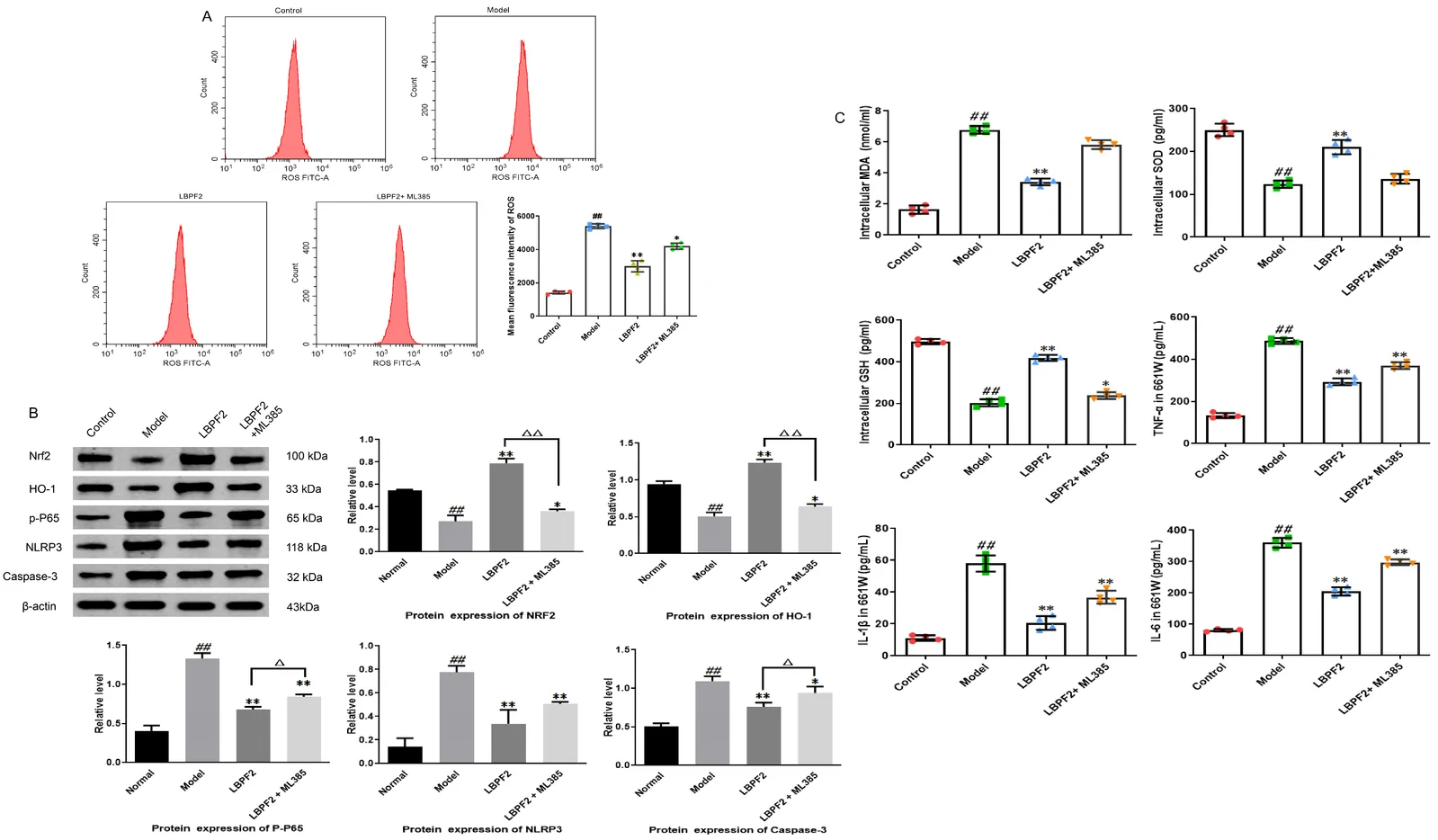
Inhibition of Nrf2 attenuates the protective effects of LBPF2 in H_2_O_2_-induced 661W cells. (**A**) Effects of LBPF2 and the Nrf2 inhibitor ML385 on cell viability and intracellular ROS levels in H_2_O_2_-treated 661W cells. (**B**) Representative Western blot images and quantitative analysis of Nrf2, HO-1, phosphorylated NF-κB (p-p65), NLRP3, and Caspase-3 protein expression in 661W cells under oxidative stress. (**C**) Effects of LBPF2 and ML385 on oxidative stress markers, including MDA, SOD, and GSH, as well as inflammatory cytokines TNF-α, IL-1β, and IL-6, determined by ELISA. Data are presented as mean ± SD (ROS assays, n = 4; Western blot, n = 3; ELISA, n = 4). Compared with the control group, ## *p* < 0.01; compared with the H_2_O_2_ model group, * *p* < 0.05 and ** *p* < 0.01; compared with the LBPF2 group, △ *p* < 0.05 and △△ *p* < 0.01.

To determine whether Nrf2 activation contributes to the cytoprotective effects of LBPF2, 661W cells were treated with LBPF2 in the presence or absence of the Nrf2 inhibitor ML385 under H_2_O_2_-induced oxidative stress conditions. LBPF2 significantly reduced intracellular ROS accumulation compared with the H_2_O_2_-treated model group. However, co-treatment with ML385 markedly attenuated these protective effects, as evidenced by increased ROS levels compared with LBPF2 treatment alone (Figure 6A).

Consistently, LBPF2 increased the expression of Nrf2 and its downstream antioxidant protein HO-1, whereas ML385 partially reversed this upregulation. Inhibition of Nrf2 also weakened the antioxidant effects of LBPF2, as indicated by increased MDA levels and reduced SOD and GSH levels (Figure 6B,C). Furthermore, ML385 restored NF-κB phosphorylation, NLRP3 expression, inflammatory cytokine production, and Caspase-3 activation compared with LBPF2 treatment alone. These findings indicate that activation of Nrf2 signaling is an important mediator of the antioxidant, anti-inflammatory, and anti-apoptotic effects of LBPF2 in oxidatively stressed 661W cells (Figure 6B,C).

### 3.7. LBPF2 Preserves Retinal Function and Morphology in rd10 Mice

**Figure 7:**
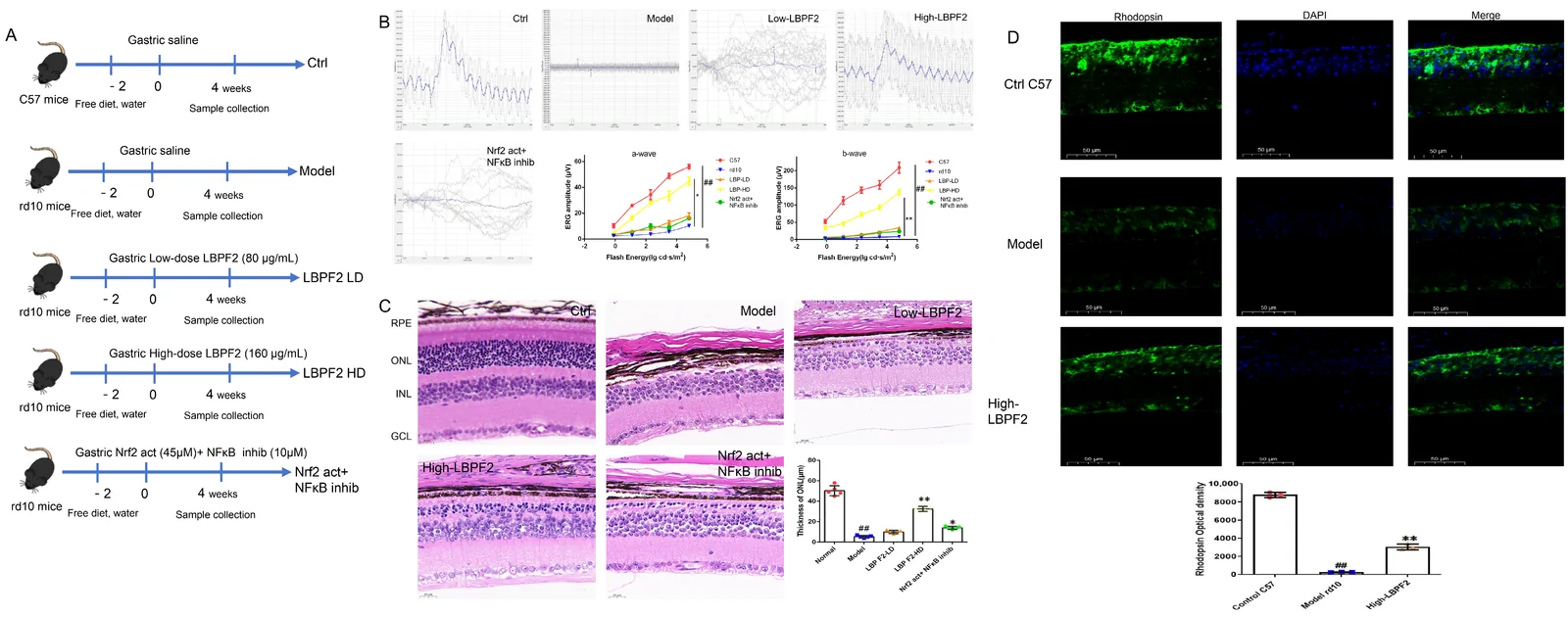
Assessment of visual function and structural changes in the mouse retina. (**A**) Schematic diagram of animal experimental design, LBPF2 was administered once daily by oral intragastric gavage at 16 or 32 μg per mouse per day, corresponding to nominal starting doses of approximately 0.80–0.89 and 1.60–1.78 mg/kg/day, respectively. (**B**) Electroretinogram showed the effect of LBPF2 on electrophysiological function; the gray lines represent background noise signals, while the blue lines represent retinal electrophysiological signals measured after noise reduction (n = 5). (**C**) H&E staining demonstrated the protective effect of LBPF2 on the retina of rd10 mice (n = 5). (**D**) Representative rhodopsin immunofluorescence images from wild-type C57BL/6J, untreated rd10 and high-dose LBPF2-treated rd10 mice, together with quantitative analysis of background-corrected rhodopsin fluorescence (n = 3). Scale bar = 50 μm. Compared to the control group, ## *p* < 0.01; compared to the model group, * *p* < 0.05, ** *p* < 0.01.

For in vivo validation, the protective effects of LBPF2 were evaluated in rd10 mice, a well-established model of inherited retinal degeneration. The experimental design is illustrated in Figure 7A.

Electroretinography (ERG) was performed to assess retinal function. Compared with wild-type mice, rd10 mice exhibited a marked reduction in both a-wave and b-wave amplitudes, indicating severe retinal dysfunction. Administration of LBPF2 significantly improved retinal electrophysiological responses, as evidenced by increased amplitudes of both ERG waves (Figure 7B).

Histological examination of chorioretinal sections further demonstrated extensive retinal degeneration in untreated rd10 mice. H&E staining revealed pronounced thinning of the outer nuclear layer (ONL), accompanied by substantial photoreceptor loss. In contrast, LBPF2 treatment effectively preserved retinal architecture and significantly increased ONL thickness. Notably, the protective effect of LBPF2 appeared more pronounced than that observed following combined treatment with the Nrf2 activator and NF-κB inhibitor (Figure 7C). To further determine whether the retained outer retinal cells expressed a photoreceptor-associated marker, rhodopsin immunofluorescence was examined. Wild-type retinas exhibited abundant rhodopsin immunoreactivity in the outer retina, whereas untreated rd10 retinas showed a marked loss and disruption of the rhodopsin-positive signal. High-dose LBPF2-treated rd10 retinas retained significantly greater rhodopsin immunoreactivity than untreated rd10 retinas, although the signal remained lower than that in wild-type mice (Figure 7D). These results support partial retention of a rod photoreceptor-associated phenotype following LBPF2 treatment.

Collectively, the ERG, H&E and rhodopsin immunofluorescence findings support partial preservation of residual photoreceptor-associated structure and retinal function rather than photoreceptor regeneration or complete functional normalization.

### 3.8. LBPF2 Attenuates Oxidative Stress and Inflammation in rd10 Retina

To determine whether the protective effects of LBPF2 observed in vivo were associated with modulation of oxidative stress and inflammatory signaling, retinal tissues were subjected to Western blot and ELISA analyses.

**Figure 8:**
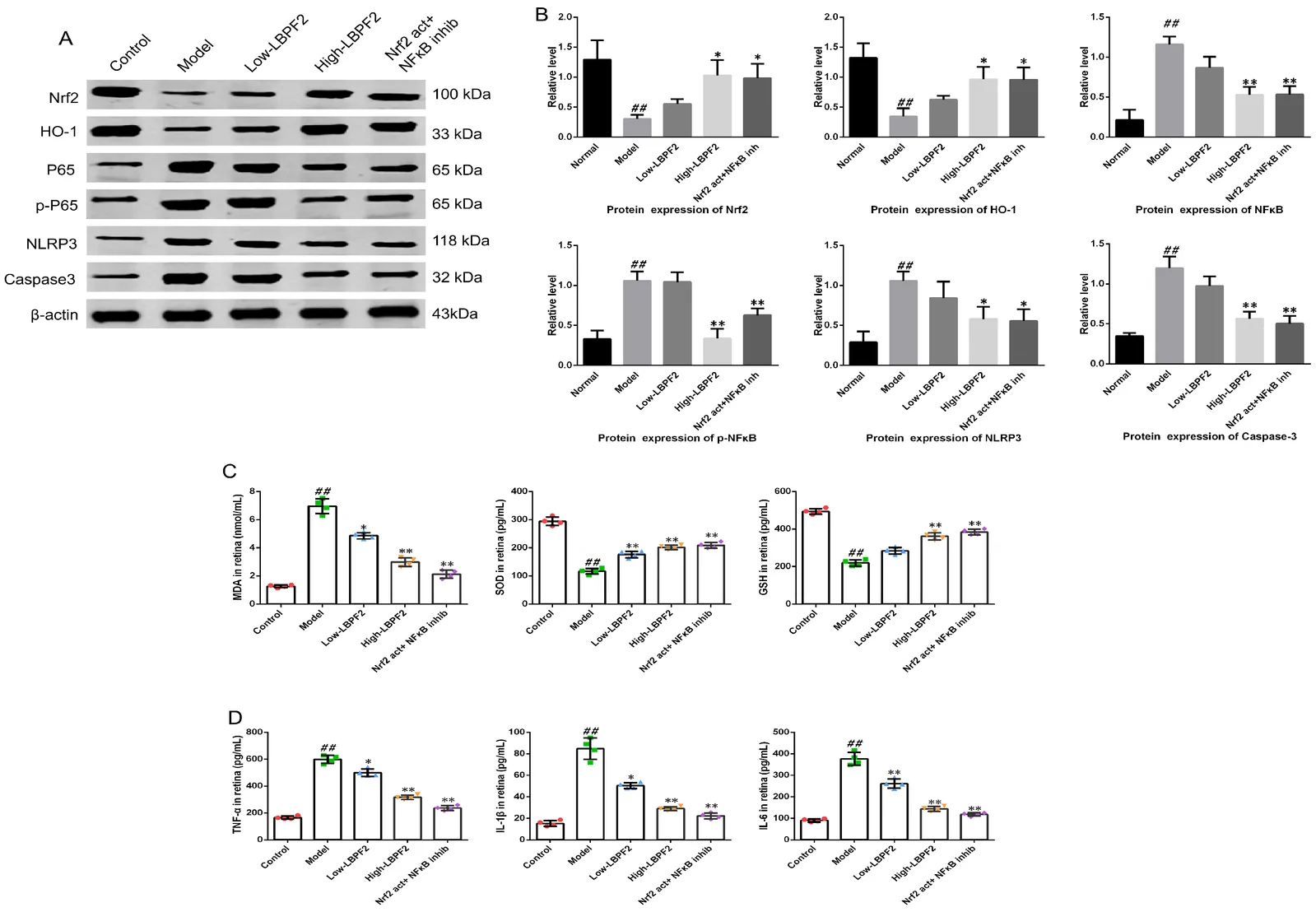
Mechanism of LBPF2 modulation of inflammation and oxidative stress in rd10 mice. (**A**,**B**) show the effects of LBPF2 on protein expression of Nrf2/HO-1, NF-κB/NLRP3, and Caspase-3 in rd10 mice (n = 3). (**C**) demonstrates that LBPF2 reduces the retinal oxidative stress marker MDA in rd10 mice, while increasing antioxidant markers SOD and GSH (n = 4). (**D**) illustrates that LBPF2 suppresses the inflammatory factors TNF-α, IL-1β, and IL-6 in the retina of rd10 mice (n = 4). Compared to the control group, ## *p* < 0.01; compared to the model group, * *p* < 0.05; ** *p* < 0.01.

Western blot results showed that retinal degeneration in rd10 mice was accompanied by significant downregulation of Nrf2 and HO-1 expression, together with increased expression of NF-κB, phosphorylated NF-κB, NLRP3, and Caspase-3. Treatment with LBPF2 significantly restored Nrf2 and HO-1 expression while suppressing activation of NF-κB/NLRP3 signaling and reducing Caspase-3 levels (Figure 8A,B).

ELISA analyses further demonstrated elevated levels of the oxidative stress marker MDA and reduced levels of the antioxidant molecules SOD and GSH in rd10 mice. LBPF2 treatment effectively reversed these alterations, resulting in a biochemical profile resembling that of wild-type animals (Figure 8C).

Similarly, retina concentrations of TNF-α, IL-1β, and IL-6 were significantly increased in rd10 mice but were markedly reduced following LBPF2 administration (Figure 8D).

Collectively, these results indicate that LBPF2 alleviates retinal degeneration by simultaneously suppressing oxidative stress and inflammation, potentially through coordinated activation of the Nrf2/HO-1 pathway and inhibition of NF-κB/NLRP3 signaling.

## 4. Discussion

The present study identifies LBPF2 as a bioactive fraction of *Lycium barbarum* polysaccharides that exerts protective effects in both oxidatively stressed 661W photoreceptor-like cells and rd10 mice. A major strength of this work is the systematic progression from fraction purification and structural characterization to biological validation in both cellular and animal models of retinal degeneration. This integrated approach is particularly important because a common limitation of studies on LBPs is the tendency to regard different preparations as functionally equivalent despite their considerable structural heterogeneity [3,5,6]. Previous studies have emphasized that physicochemical differences among polysaccharide fractions are biologically meaningful and that detailed structural characterization is essential for accurately interpreting the biological activities of LBP-derived products [3,5,9].

The structural characteristics of LBPF2 are likely closely associated with its biological activity. In the present study, LBPF2 was identified as a glucose-rich polysaccharide fraction with a relatively narrow molecular weight distribution centered at approximately 41 kDa. Structural analyses further demonstrated that LBPF2 primarily consists of →4)-α-D-Glcp-(1→ and →4,6)-α-D-Glcp-(1→ backbone residues, with side chains containing α-D-Glcp-(1→ and →6)-β-D-Glcp-(1→ units. Although the current study does not establish a direct causal relationship between any individual structural motif and retinal protection, our findings demonstrate that this relatively homogeneous glucan-rich fraction exhibits substantially greater biological activity compared to a parallel fraction isolated from the same crude LBP preparation. This observation is consistent with previous studies showing that molecular weight, branching degree, monosaccharide composition, and glycosidic linkage patterns can profoundly influence the antioxidant and immunomodulatory properties of polysaccharides [33,34,35]. Consequently, the present findings extend the current understanding of LBP bioactivity beyond the conventional crude-extract paradigm and support a more precise fraction-based concept of food-derived functionality.

Because LBPF2 is predominantly composed of glucose residues, a potential nutritional contribution to cellular metabolism should be considered. Nevertheless, the glucose-rich composition was determined after hydrolysis and should not be equated with the presence of freely available glucose. At the highest concentration tested, LBPF2 supplied approximately 71.2 μg/mL of glucose-residue equivalents, which was substantially lower than the basal glucose concentration of the culture medium. Furthermore, the coordinated modulation of ROS, apoptosis, inflammatory mediators and Nrf2-dependent signaling suggests that its effects cannot be attributed solely to increased energy-substrate availability. Future studies incorporating free-glucose controls, polysaccharide uptake and degradation assays, and measurements of ATP production and glycolytic flux will be required to distinguish signaling-mediated effects from possible metabolic contributions.

The concentration-screening findings also indicate that LBPF2 did not produce a simple linear concentration–response relationship. Although 160 μg/mL was generally more effective than 80 μg/mL within the selected non-cytotoxic range, exposure to 320 μg/mL markedly reduced cell viability. This pattern suggests the presence of an upper tolerability limit and potentially a non-linear or inverted-U-shaped response. Therefore, 160 μg/mL should be regarded as the most effective concentration among those tested rather than as evidence that progressively higher LBPF2 concentrations necessarily produce greater protection. Additional concentrations within and above the 80–160 μg/mL range will be required to define the optimal therapeutic window more precisely.

Another important aspect of this study is the integrated mechanistic framework linking oxidative stress and inflammatory signaling. In H_2_O_2_-induced 661W cells, LBPF2 improved antioxidant capacity, reduced lipid peroxidation, and attenuated inflammatory responses. The observed changes in MDA, SOD, and GSH further support the antioxidant activity of LBPF2. MDA is a well-established end-product of lipid peroxidation and is widely used as a biomarker of oxidative damage in retinal degeneration and other neurodegenerative disorders [12,15]. Elevated MDA levels indicate increased oxidative injury to cellular membranes, whereas reduced MDA levels following LBPF2 treatment suggest attenuation of lipid peroxidation. In contrast, SOD and GSH represent key components of the endogenous antioxidant defense system. SOD catalyzes the conversion of superoxide radicals into less reactive species, while GSH functions as a major intracellular redox buffer and detoxifying molecule [18,19]. Restoration of SOD and GSH levels by LBPF2 therefore indicates improved antioxidant capacity and maintenance of cellular redox homeostasis. Similar changes in MDA, SOD, and GSH have been associated with retinal protection in experimental models of retinal degeneration and oxidative stress-induced retinal injury [25,36,37]. The coordinated reduction of MDA together with restoration of SOD and GSH levels suggests that LBPF2 not only suppresses oxidative damage but may also enhance endogenous antioxidant defense mechanisms, which is consistent with the observed activation of Nrf2/HO-1 signaling.

Similarly, in rd10 mice, LBPF2 treatment improved retinal function, preserved retinal morphology, and modulated oxidative and inflammatory biomarkers in a manner consistent with retinal protection. These findings suggest that LBPF2 acts not merely as a direct radical scavenger but rather as a regulator of cellular stress adaptation through coordinated modulation of redox and inflammatory signaling pathways.

Oxidative stress has been widely recognized as a major contributor to cone photoreceptor degeneration and disease progression in RP [1,12,13]. Secondary cone degeneration is a major determinant of irreversible central vision loss in patients with RP, and increasing evidence suggests that excessive oxidative stress plays a central role in this process [1,13]. In the present study, H_2_O_2_-induced 661W cells, a well-established cone photoreceptor-like cell model38, were used to mimic oxidative injury to cone photoreceptors. The observed reduction in intracellular ROS, restoration of antioxidant capacity, and suppression of apoptosis following LBPF2 treatment therefore support the hypothesis that LBPF2 may protect cone photoreceptors against oxidative stress-associated degeneration. Because preservation of cone photoreceptors is directly associated with maintenance of daylight and central vision in RP, the protective effects observed in 661W cells further support the translational relevance of LBPF2. These cellular findings were further corroborated by the in vivo rd10 mouse model, in which LBPF2 preserved retinal structure and function.

Likewise, chronic inflammation, microglial activation, and NLRP3 inflammasome signaling have been implicated in the amplification of retinal injury [21,38]. Increasing evidence indicates that non-genetic pathogenic processes, including oxidative stress and innate immune activation, can substantially influence the progression of retinal degeneration, even when the initiating insult is caused by a mutation-specific defect [39,40]. The present findings support this concept by demonstrating that modulation of these shared pathological pathways is associated with improved retinal outcomes.

The current results are also consistent with previous reports investigating the retinal protective effects of goji berry-derived polysaccharides. For instance, Tang et al. demonstrated that LBP attenuated photoreceptor degeneration in a light-induced retinal injury model [25], whereas Liu et al. reported that LBP improved retinal morphology and visual function in rd1 mice [26]. These earlier studies evaluated crude or broadly defined LBP preparations; therefore, their retinal effects could not be attributed to a chromatographically separated and structurally characterized polysaccharide fraction. In contrast, the present study isolated LBPF2 by DEAE-cellulose chromatography and characterized it as a glucose-rich fraction with 81.1% total carbohydrate content, an average molecular weight of approximately 41 kDa, and a narrow molar-mass distribution (Mw/Mn = 1.022). The present study advances this field by chemically defining the active fraction of LBPF2 and simultaneously evaluating oxidative and inflammatory responses within a unified experimental framework. Our findings strengthen the emerging concept that retinal protection is associated not simply with the presence of LBP as a broad category of natural products, but rather with specific fraction-level structural and functional properties that warrant further investigation.

The in vivo component of this study further enhances its translational relevance. The 661W cell line is widely used as an experimental model for investigating cone photoreceptor stress and degeneration [41], whereas rd10 mice are considered a valuable model for therapeutic intervention studies as retinal degeneration develops more gradually compared to rd1 mice [27,36]. Previous studies have established a close relationship between structural retinal degeneration and declining ERG responses in rd10 mice [27]. Within this context, the present findings demonstrate that LBPF2 significantly improves retinal electrophysiological function and preserves retinal morphology, supporting the physiological relevance of LBPF2-mediated protection; however, the causal role of Nrf2 in vivo remains to be determined. More broadly, our results are consistent with previous studies showing that antioxidant-based interventions can delay photoreceptor degeneration in RP models [37].

Importantly, the higher ERG amplitudes, greater ONL thickness and increased rhodopsin immunoreactivity observed after LBPF2 treatment should not be interpreted as evidence of photoreceptor regeneration. The ERG a-wave provides information related predominantly to photoreceptor function, whereas the b-wave reflects post-receptoral retinal activity; neither measurement demonstrates the generation of new cells. Rhodopsin immunofluorescence further supports retention of rod photoreceptor-associated material in LBPF2-treated retinas. The most conservative interpretation is therefore that LBPF2 attenuated ongoing degeneration and partially preserved residual photoreceptors and retinal circuitry. Because no proliferative labeling, lineage tracing or ultrastructural analysis was performed, de novo photoreceptor generation and restoration of normal outer-segment ultrastructure were not demonstrated.

Based on body-surface-area normalization, the pharmacologically active LBPF2 doses used in mice corresponded to approximate human-equivalent doses of 0.065–0.072 and 0.130–0.144 mg/kg/day, equivalent to approximately 3.9–4.3 and 7.8–8.6 mg/day for a 60-kg adult. These calculations provide a preliminary scale for interpreting the experimental doses but should not be regarded as recommended clinical doses. Body-surface-area conversion does not account for potential interspecies differences in gastrointestinal degradation, oral bioavailability, microbiota-mediated metabolism or systemic exposure. Pharmacokinetic studies, repeated-dose toxicology and determination of a no-observed-adverse-effect level will therefore be required before a human starting dose can be proposed.

Several human studies provide preliminary clinical context for the oral use of L. barbarum preparations. In a double-masked, placebo-controlled study involving patients with RP, 12 months of oral L. barbarum granules was associated with preservation of visual acuity and macular thickness, although no significant between-group differences were detected in full-field ERG parameters [42]. In patients with early AMD, consumption of 25 g of goji berries daily for 90 days increased serum zeaxanthin and macular pigment optical density [43]. Similarly, supplementation with 13.7 g/day of a milk-based goji formulation for 90 days increased plasma zeaxanthin and antioxidant capacity and was associated with stabilization of selected macular characteristics in older adults [44]. Standardized goji juice has also been reported to improve circulating antioxidant biomarkers in healthy participants [45]. These clinical observations support the oral feasibility and translational interest of goji-derived products. Nevertheless, these preparations contained multiple bioactive constituents and were not chemically equivalent to LBPF2. Consequently, they do not establish the clinical efficacy, optimal dose, or long-term safety of the structurally characterized fraction investigated here.

**Figure 9:**
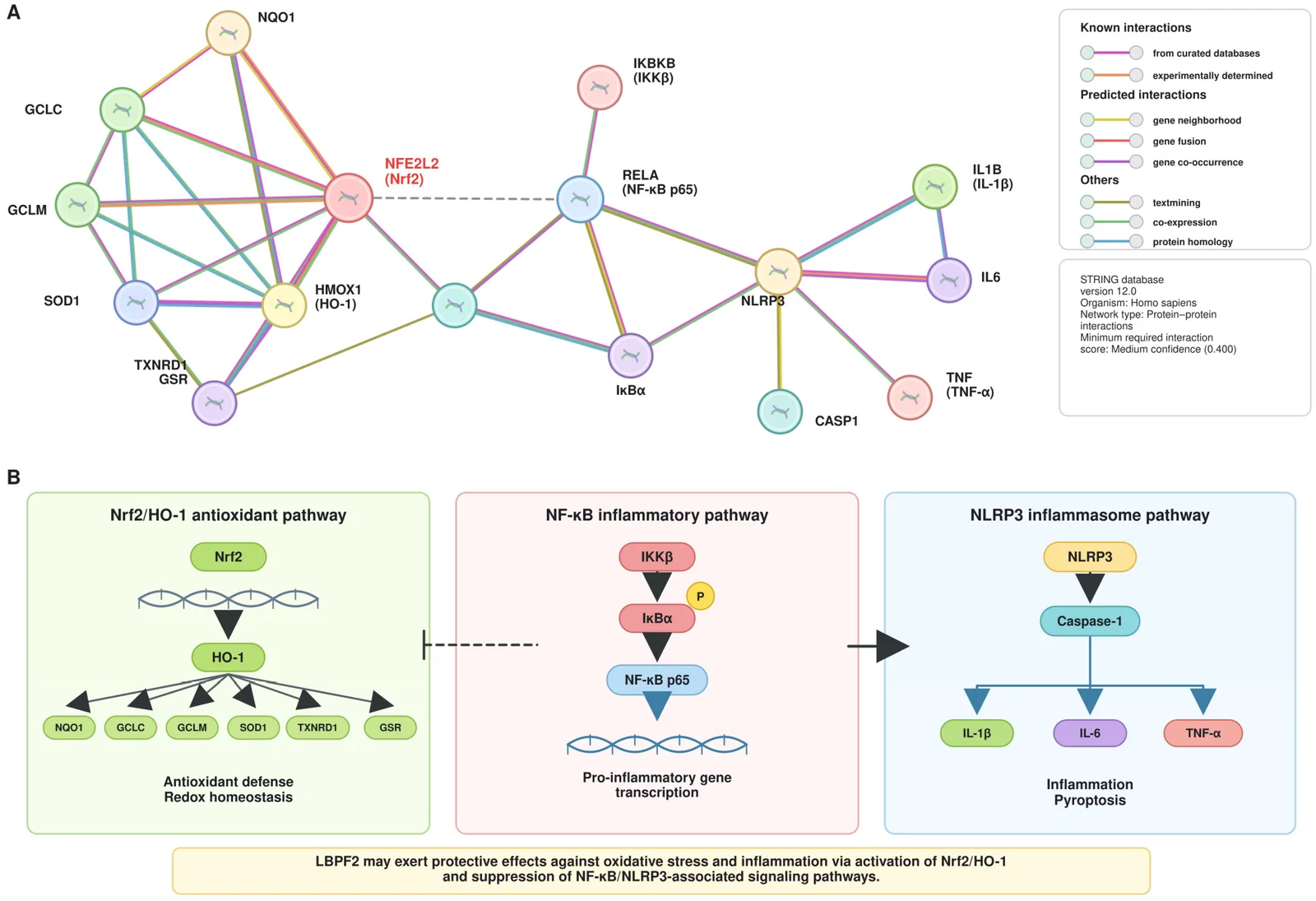
Protein–protein interaction network and proposed signaling mechanisms underlying the protective effects of LBPF2. (**A**) Protein–protein interaction (PPI) network generated using the STRING database (version 12.0), illustrating the relationships between antioxidant and inflammation-related proteins associated with Nrf2/HO-1 and NF-κB/NLRP3 signaling pathways. NFE2L2 (Nrf2), HMOX1 (HO-1), NQO1, GCLC, GCLM, SOD1, TXNRD1, and GSR form a tightly connected antioxidant defense network, whereas RELA (NF-κB p65), IKBKB, NLRP3, CASP1, TNF, IL1B, and IL6 constitute a closely linked inflammatory signaling module. The interaction network suggests extensive crosstalk between oxidative stress and inflammatory pathways. (**B**) Proposed mechanisms of action of LBPF2 in retinal degeneration. Activation of the Nrf2/HO-1 pathway enhances antioxidant defenses and maintains redox homeostasis through upregulation of downstream antioxidant enzymes. Concurrently, suppression of NF-κB signaling inhibits the activation of the NLRP3 inflammasome and reduces the production of pro-inflammatory cytokines, including TNF-α, IL-1β, and IL-6. Collectively, these mechanisms may contribute to the protective effects of LBPF2 against oxidative stress, inflammation, and apoptosis-associated retinal degeneration.The grey dashed line between NFE2L2 (Nrf2) and RELA (NF-κB p65) indicates functional crosstalk or an indirect regulatory association rather than a direct protein–protein interaction retrieved from STRING.

Several limitations of this study should be acknowledged. First, although the present findings support the involvement of Nrf2/HO-1 activation and suppression of NF-κB/NLRP3 signaling, the direct molecular target of LBPF2 remain unidentified. Interestingly, growing evidence suggests an extensive crosstalk between Nrf2 and NF-κB signaling. For example, activation of Nrf2 can suppress NF-κB-dependent inflammatory responses through multiple mechanisms, whereas persistent NF-κB activation may impair antioxidant defenses [46]. The STRING interaction network generated in the present study further supports the existence of a closely connected regulatory network linking oxidative stress and inflammatory signaling pathways (Figure 9), identifying candidate target molecules that can be biochemically studied in the future. Therefore, the anti-inflammatory effects of LBPF2 may arise, at least in part, secondary to enhancement of Nrf2-mediated antioxidant defenses. Second, despite the detailed structural characterization of LBPF2, the specific structural determinants responsible for its biological activity remain unclear. Future studies involving controlled depolymerization, selective side-chain modification, receptor-blocking experiments, and comparisons with additional purified fractions will be necessary to establish structure–activity relationships more definitively. Third, orally administered polysaccharides may undergo gastrointestinal digestion, biotransformation, or microbiota-mediated metabolism before reaching systemic circulation, potentially altering their structural characteristics and biological activities prior to exerting systemic effects [47,48]. Consequently, the active entity responsible for retinal protection in vivo may differ from the native LBPF2 fraction characterized in vitro. Although glucose-rich polysaccharides should not be equated with free dietary glucose, the digestibility and glycemic effects of LBPF2 remain unknown. Fasting glucose, insulin, oral glucose tolerance and glycated hemoglobin were not evaluated in the present study. These endpoints, together with long-term metabolic safety, should be assessed before clinical use, particularly in individuals with diabetes. Despite these limitations, the present work provides a robust ingredient-level framework that extends beyond conventional crude-extract studies and supports LBPF2 as a promising food-derived candidate for mitigating oxidative stress and inflammation-associated retinal degeneration.

## 5. Conclusions

In conclusion, LBPF2 is a structurally defined and biologically active polysaccharide fraction isolated from *Lycium barbarum*. In both cellular and animal models of retinal degeneration, LBPF2 protected against oxidative and inflammatory injury through mechanisms associated with activation of the Nrf2/HO-1 pathway and suppression of NF-κB/NLRP3 signaling. By integrating fraction screening, structural elucidation, and mechanism-oriented biological validation, this study strengthens the link between polysaccharide structure and biological function and provides further support for the development of structurally defined goji berry-derived ingredients as potential therapeutic or functional food candidates for retinal degenerative diseases [49,50]. Further oral bioavailability, pharmacokinetic, metabolic safety and dose-ranging studies are required before a clinically appropriate dose of LBPF2 can be established.

## Figures and Tables

**Table 1:** Purity of *Lycium barbarum* polysaccharides.

Sample Name (Number of Tubes)	Sample Weight (mg)	Absorbance Mean Value	Total LBP (mg)	Purity Rate
LBP-Fraction 1 (7–19)	25.1	0.188	21.09	84.0%
LBP-Fraction 2 (28–40)	24.8	0.183	20.12	81.1%

**Table 2:** HPAEC analysis of monosaccharides in LBPF2.

Number	Peak Name	Retention Time (min)	Peak Area (nC × min)	Peak Elevation (nC)	Content (μg/mg)
1	n.a.	1.409	9.219	118.767	n.a.
2	n.a.	1.817	1.027	4.994	n.a.
3	Fuc	4.625	0.312	2.147	2.82
4	Ara	10.284	0.831	3.119	8.95
5	Gal	13.300	0.780	2.097	8.34
6	Glc	15.109	42.183	70.234	445.16
7	n.a.	30.175	2.465	83.051	n.a.

n.a., not available.

**Table 3:** Methylation analysis of LBPF2.

Derivatives Name	Linking Type	RT (min)	Mass Fragments (*m*/*z*)	Mw	Relative Molar Ratio (%)
1,5-di-O-acetyl-2,3,4,6-tetra-O-methyl glucitol	t-Glc(p)	9.508	87,102,118,129, 145,161,162,205	323	22.94
1,3,5-tri-O-acetyl-2,4,6-tri-O-methyl glucitol	3-Glc(p)	12.847	87,101,118,129, 161,202,234	351	2.99
1,2,5-tri-O-acetyl-3,4,6-tri-O-methyl glucitol	2-Glc(p)	13.203	88,101,129,130, 161,190,205	351	2.34
1,5,6-tri-O-acetyl-2,3,4-tri-O-methyl glucitol	6-Glc(p)	14.441	87,99,102,118,12 9,162,189,233	351	11.82
1,4,5-tri-O-acetyl-2,3,6-tri-O-methyl glucitol	4-Glc(p)	14.790	87,102,113,118, 129,162,233	351	43.05
1,3,4,5-tetra-O-acetyl-2,6-di-O-methyl glucitol	3,4-Glc(p)	16.980	87,118,129,143, 185,203,305	379	1.50
1,2,3,5-tetra-O-acetyl-4,6-di-O-methyl glucitol	2,3-Glc(p)	17.288	86,87,101,129,16 1,202,262	379	1.13
1,2,4,5-tetra-O-acetyl-3,6-di-O-methyl glucitol	2,4-Glc(p)	17.651	87,88,99,113,130, 190,233	379	1.35
1,3,5,6-tetra-O-acetyl-2,4-di-O-methyl glucitol	3,6-Glc(p)	18.527	87,101,118,129, 189,202,234	379	2.03
1,4,5,6-tetra-O-acetyl-2,3-di-O-methyl glucitol	4,6-Glc(p)	19.095	85,102,118,127, 159,162,201,261	379	5.73
1,2,5,6-tetra-O-acetyl-3,4-di-O-methyl glucitol	2,6-Glc(p)	19.191	87,88,99,100,129, 130,189,190	379	1.67
1,3,5,6-tetra-O-acetyl-2,4-di-O-methyl galactitol	3,6-Gal(p)	19.704	87,101,118,129, 160,189,234	379	1.04
1,3,4,5,6-penta-O-acetyl-2-O-methyl galactitol	3,4,6-Gal (p)	21.510	97,118,129,139, 160,333	407	1.33
1,2,4,5,6-penta-O-acetyl-3-O-methyl glucitol	2,4,6-Glc (p)	22.373	71,88,99,117,127, 130,137,190,261, 338	407	1.08

RT, Retention time; Mw, Molecular weight.

**Table 4:** presents the assignment of 1H and 13C chemical shifts for sugar residues.

Code	Glycosyl Residues	Chemical Shifts (ppm)					
		H1/C1	H2/C2	H3/C3	H4/C4	H5/C5	H6/C6
A	→4)-α-D-Glcp-(1→	5.33 99.57	3.55 71.49	3.89 73.31	3.58 76.68	3.76 71.14	3.77 60.41
B	α-D-Glcp-(1→	4.9 97.81	3.49 71.7	3.66 72.84	3.36 69.47	3.92 73.12	3.55 61.05
C	→6)-β-D-Glcp-(1→	4.45 102.74	3.25 73.08	3.43 75.62	3.57 71.98	3.78 72.68	3.91 65.5
D	→4,6)-α-D-Glcp-(1→	5.31 99.73	3.56 71.76	3.89 70.22	3.77 76.32	3.84 73.97	3.8 68.44

## Data Availability

The data supporting the findings of this study are available from the author upon reasonable request.

## Abbreviations

The following abbreviations are used in this manuscript:

CCK-8	Cell Counting Kit-8
COSY	Correlation spectroscopy
DEAE	Diethylaminoethyl
DEPT	Distortionless enhancement by polarization transfer
ELISA	Enzyme-linked immunosorbent assay
ERG	Electroretinography
Fru	Fructose
Fuc	Fucose
Gal	Galactose
Gal-UA	Galacturonic acid
GC-MS	Gas chromatography-mass spectrometry
Glc	Glucose
Glc-UA	Glucuronic acid
GSH	Glutathione
Gul-UA	Guluronic acid
H&E	Hematoxylin and eosin
H_2_O_2_	Hydrogen peroxide
HMBC	Heteronuclear multiple-bond correlation
HO-1	Heme oxygenase-1
HPAEC	High-performance anion-exchange chromatography
HSQC	Heteronuclear single-quantum coherence
IL-1β	Interleukin-1 beta
IL-6	Interleukin-6
LBP	Lycium barbarum polysaccharide(s)
LBPF1	Lycium barbarum polysaccharide fraction 1
LBPF2	Lycium barbarum polysaccharide fraction 2
Man	Mannose
Man-UA	Mannuronic acid
MDA	Malondialdehyde
Mn	Number-average molecular weight
Mp	Peak molecular weight
Mw	Weight-average molecular weight
Mz	z-average molecular weight
NF-κB	Nuclear factor kappa B
NLRP3	NOD-like receptor pyrin domain-containing 3
NMR	Nuclear magnetic resonance
NOESY	Nuclear Overhauser effect spectroscopy
Nrf2	Nuclear factor erythroid 2-related factor 2
ONL	Outer nuclear layer
PPI	Protein–protein interaction
Rha	Rhamnose
Rib	Ribose
ROS	Reactive oxygen species
RP	Retinitis pigmentosa
SEC-MALLS-RI	Size-exclusion chromatography coupled with multi-angle laser light scattering and refractive index detection
SEM	Scanning electron microscopy
SOD	Superoxide dismutase
STRING	Search Tool for the Retrieval of Interacting Genes/Proteins
TNF-α	Tumor necrosis factor-alpha
TOCSY	Total correlation spectroscopy
WB	Western blotting
Xyl	Xylose
